# Impact of Body Mass Index on Surgical and Oncological Outcomes in Laparoscopic Total Mesorectal Excision for Locally Advanced Rectal Cancer after Neoadjuvant 5-Fluorouracil-Based Chemoradiotherapy

**DOI:** 10.1155/2017/1509140

**Published:** 2017-09-14

**Authors:** Yanwu Sun, Pan Chi

**Affiliations:** Department of Colorectal Surgery, Fujian Medical University Union Hospital, Fuzhou, Fujian, China

## Abstract

**Aims:**

To evaluate the impact of body mass index (BMI) on the surgical outcome of laparoscopic total mesorectal excision (laTME) for locally advanced rectal cancer (LARC, clinically staged as UICC stage II/III) after neoadjuvant chemoradiotherapy (nCRT).

**Methods:**

312 LARC patients undergoing laTME after nCRT were divided into nonobese (BMI < 25.0 kg/m^2^, *n* = 249) and obese (BMI ≥ 25.0 kg/m^2^, *n* = 63) groups. Preoperative radiotherapy was delivered in 45–50.4 Gy/25f, 5 days/week, and concurrent chemotherapy using FOLFOX or CapeOX. Technical feasibility, postoperative and oncological outcome were compared between groups.

**Results:**

Obese patients had significantly longer operative time (*P* = 0.004). There was no significant difference regarding estimated blood loss, conversion, postoperative recovery, and morbidities. Multivariate analysis demonstrated that higher ASA score and abdominoperineal resection were risk factors for postoperative complications and diverting stoma was a protective factor. The length of resection margin, circumferential resection margin involvement, and number of lymph node retrieved were comparable. With a median follow-up time of 55 months (ranging 20–102 months), oncological outcome was comparable in terms of overall survival, local recurrence, and distant metastasis.

**Conclusions:**

Obesity does not affect surgical or oncological outcome of laTME after nCRT. LaTME may be feasible and safe to obese LARC patients after nCRT in a specialized center.

## 1. Introduction

Obesity is a major public health problem associated with numerous morbidities [1]. Body mass index (BMI), the basis for the assessment of obesity, has been shown to be a risk factor for colorectal cancer incidence and death [1, 2]. It was recognized that higher BMI was associated with increased technical difficulties and postoperative morbidity during colorectal surgery [3–5]. In contrast, other studies reported no negative impact of obesity on surgical outcome after rectal surgery [6, 7].

The concept of total mesorectal excision (TME), introduced by Professor Bill Heald (father of TME) [8], significantly reduces the rate of local recurrence of rectal cancer. Recent randomized clinical trials (RCTs) have demonstrated better results for laparoscopic TME (laTME) in terms of short- and long-term oncological outcomes, when compared with open TME [9–11]. However, obesity presents both negative and positive challenges to laparoscopic rectal surgery: the laparoscopic procedure becomes more technically demanding; on the other hand, the laparoscopic approach may be particularly advantageous to obese patients, resulting in reduced postoperative pain, faster postoperative recovery, and shorter hospital stay [5].

The long-term results of the CAO/ARO/AIO-94 trial of the GRCSG have demonstrated the enormous effect of neoadjuvant chemoradiotherapy (nCRT) [12]. Nowadays, nCRT followed by total mesorectal excision (TME) has become the standard of care for patients with locally advanced rectal cancer (LARC), clinically staged as UICC stages II and III (cUICC—II/III) [12, 13]. A phase 3 study (CAO/ARO/AIO-04), which integrated oxaliplatin into standard fluorouracil-based combined modality treatment, has reported that intensification of CRT was feasible and led to a higher pathologically complete (pCR) response [14–16]. Surgeons may encounter increased technical difficulties and surgical morbidity during laTME following nCRT [17]. Therefore, implementation of laTME following nCRT in those obese patients requires a specific assessment of the impact of obesity on this invasive procedure. To date, however, there have been few studies looking at the impact of obesity on surgical outcomes in laTME for rectal cancer following nCRT.

This study was intended to evaluate the impact of obesity, as measured by BMI, on feasibility, safety, and oncological outcome of laTME for LARC patients after nCRT in a high-volume center. We also sought to identify factors associated with postoperative morbidity of laTME following nCRT in the present study.

## 2. Materials and Methods

### 2.1. Patient Population

This study was a retrospective and monocentric analyses. Between 2008 and 2014, 312 consecutive patients with LARC who underwent laTME were identified from our prospectively maintained database. The inclusion criteria were as follows: (1) clinically staged as UICC stages II and III, (2) within 12 cm above the anal verge, (3) a histologically proven adenocarcinoma, and (4) no evidence of distant metastasis. Patients with previous or concurrent malignancies and those who underwent emergent surgery, palliative resection, or local excision were excluded. Our institutional review board approved this study.

### 2.2. Neoadjuvant Chemoradiotherapy

Patients chose inclusion to the direct surgery or nCRT groups based on the current stage of their disease and after understanding the risks and benefits and without the influence of the surgeon. All patients underwent CT simulation of the three-field technique for conformal radiotherapy planning. Clinical target volume (CTV) included the primary tumor, the mesentery with vascular supply, and the perirectal, presacral, and internal iliac nodes up to the S1/S2 junction. Planning target volume (PTV) was formed by enlarging 10 to 15 mm on the basis of the clinical target volume. Preoperative radiotherapy was delivered in fractions of 1.8–2.0 Gy, 5 fractions per week for 5-6 consecutive weeks followed by a boost of 5.4 Gy to reach a dose of 45–50.5 Gy. The chemotherapeutic regimens with dosages were as follows: FOLFOX4: oxaliplatin 85 mg/m^2^ IV, day 1, leucovorin 200 mg/m^2^ IV × 2 days, 5-FU 400 mg/m^2^ IV bolus × 2 days, then 600 mg/m^2^/d × 2 days as a 22-hour continuous infusion. Repeat every 2 weeks to a total of 6 months of perioperative therapy [14, 15]. CapeOX: oxaliplatin 130 mg/m^2^ IV, day 1, capecitabine 1000 mg/m^2^ twice daily, days 1–14 every 3 weeks. Repeat every 3 weeks to a total of 6 months of perioperative therapy [18].

### 2.3. Surgical Procedure

**Figure 1:**
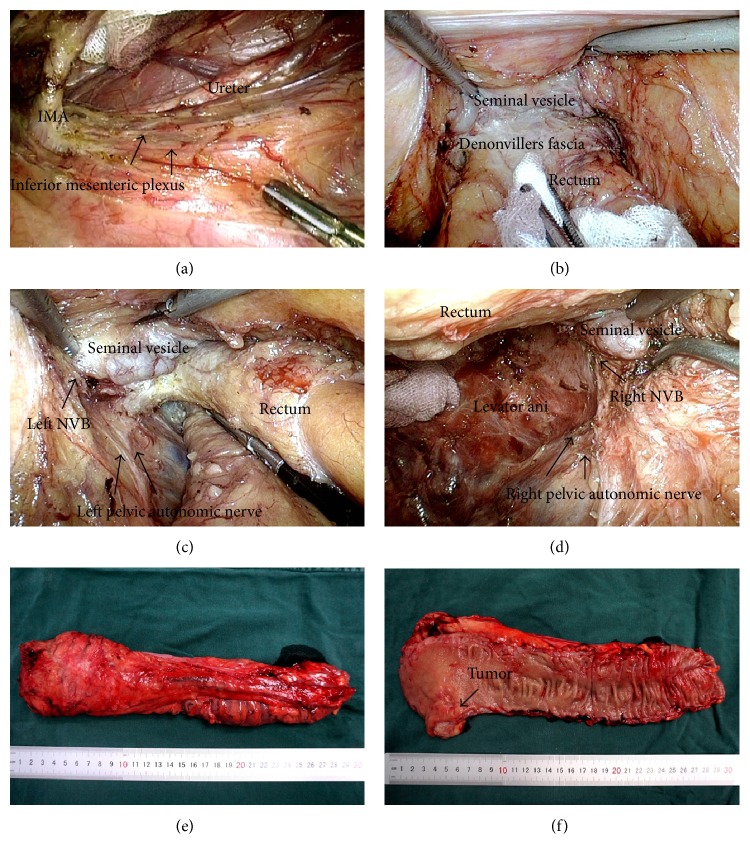
Pictures from laparoscopic surgery in obese patients with locally advanced rectal cancer. (a) A medial to lateral dissection was performed, the IMA was skeletonized and ligated, the inferior mesenteric plexus was identified and protected; (b) the anterior space of rectum was well exposed, and the anterior layer of Denonvilliers' fascia was dissected below the seminal vesicle; (c) the left “holy plane” was well exposed, the left NVB and pelvic autonomic nerve were identified and protected; (d) the right “holy plane” was well exposed, the right NVB and pelvic autonomic nerve were identified and protected; (e) the specimen with an intact mesorectum fascia; (f) the tumor was downsized after nCRT (tumor size: pre-CRT 2.5 cm, post-CRT 1.5 cm) with a distal resection margin of more than 2 cm; IMA: inferior mesenteric artery; NVB: neurovascular bundle.

Procedures were performed by the same surgical team. Our team has experience with more than 3000 cases of laparoscopic colorectal surgeries since September, 2000 [19]. Surgery was performed 6–8 weeks after the end of radiation. Laparoscopic rectal surgery was standardized in our center, as presented in Figure 1(). TME was performed for middle and low rectal cancers, and partial TME with a distal margin of 5 cm was performed for high rectal cancers. The inferior mesenteric artery (IMA) was ligated at the level of the root to ensure a tension-free anastomosis, and IMA lymph nodes were dissected to the IMA just below the bifurcation of the left colic artery. Pelvic autonomic nerves were identified and preserved. After the dissection was completed, the rectum was transected with an endoscopic linear stapler. A 5-6 cm Pfannenstiel incision was made for specimen extraction and proximal transection. An end-to-end anastomosis was constructed using a circular stapler, and the donuts were checked. Air leak test was used to identify mechanically insufficient anastomosis. Generally, protective diverting ileostomy was performed in an effort to protect low rectal anastomosis, taking into considerations the general health of the patient, nutritional status, diabetes, the distance of the anastomosis from the anal verge, and the use of nCRT. Starting approximately 3 to 4 weeks after surgery, patients received adjuvant chemotherapy for 6 months. Two different chemotherapy regimens were used, including FOLFOX or CapeOX.

### 2.4. Follow-Up

Follow-up evaluations were performed every 3 months for the first 3 years, then every 6 months for the next 2 years, and annually thereafter. At each visit, a physical examination, serum carcinoembryonic antigen (CEA) test, chest X-ray or computed tomography (CT) scans, and abdominopelvic magnetic resonance imaging (MRI) or CT scans were performed. A colonoscopy was performed annually after surgery. Positron emission tomography (PET) examination was added when needed.

### 2.5. Definitions

Using the proposed International Obesity Task Force (IOTF) classifications for obesity in Asians, BMI cut-off points were used to categorize patients into two groups: nonobese (BMI < 25.0 kg/m^2^) and obese (BMI ≥ 25.0 kg/m^2^) [20]. CRM was measured using a microscopic ruler, and CRM involvement was defined as a microscopic tumor <1 mm from the circumferential or radial resection margins [21]. Tumor regression levels were graded according to the Rectal Cancer Regression Grade (RCRG) method by Wheeler et al. [22] as follows: RCRG 1, sterilization or only microscopic foci of adenocarcinoma remaining, with marked fibrosis; RCRG 2, marked fibrosis but macroscopic disease present; RCRG 3, little or no fibrosis, with abundant macroscopic disease. Postoperative morbidity was defined as any complication occurring within 30 days after surgery and was graded according to the Clavien-Dindo classification [23]. Major morbidity was defined as any event that required endoscopic, radiological, surgical reoperations or intensive care unit treatment (Clavien-Dindo grades III-IV) and minor morbidity as Clavien-Dindo grades I-II. Perioperative mortality was defined as any death either within 30 days after surgery or during the hospitalization period.

### 2.6. Statistical Analysis

Statistical analysis was performed using SPSS version 20.0 (SPSS INC., Chicago). The categorical variables were expressed as numbers with percentages and compared using a chi-square test or Fisher's exact test when appropriate. Normally distributed data were described as the means ± standard deviations and analyzed with Student's *t*-tests. Nonnormally distributed data were presented with medians and ranges and analyzed with the Mann–Whitney *U* test. Univariate and multivariate logistic analysis were performed to identify risk factors of postoperative complications. Overall survival (OS) was measured from the date of surgery to the date of death from any cause [24, 25]. Local recurrence was defined as any tumor relapse within the pelvis, perineum, or anastomosis as diagnosed by pathological examination. Distant metastasis was identified as evidence of a tumor in any other area diagnosed by imagining or pathological examinations. Survival outcomes were assessed using the Kaplan-Meier method and log-rank test. Statistical significance was defined as *P* < 0.05.

## 3. Results

### 3.1. Study Population

A total of 312 LARC patients who underwent laTME after nCRT were included in our study (nonobese, 249; obese, 63). The median BMI of the nonobese patients was 21.7 kg/m^2^, significantly lower than 26.9 kg/m^2^ in the obese group (*P* < 0.001). No significant differences were observed in clinical characteristics between two groups, as summarized in Table 1.

### 3.2. Technical Feasibility

Obese patients had significantly longer operative time (224.3 ± 38.8 min versus 207.9 ± 43.9 min, *P* = 0.004), as summarized in Table 2. A trend towards greater estimated blood loss was observed among obese patients (73.6 ± 59.1 ml versus 63.2 ± 87.6 ml), but it was not statistically significant (*P* = 0.265). Conversion to open procedure occurred in 9 nonobese patients and 4 obese patients, and the difference was not significant (3.6% versus 6.3%, *P* = 0.332). Conversions in nonobese patients were due to severe intra-abdominal adhesions (three), abdominal bleeding (one), bowel injury (one), fixed tumor (two), and a narrow pelvis (two). The reasons for conversion in obese patients were severe intraabdominal adhesions (two) and a narrow pelvis (two).

### 3.3. Postoperative Recovery

No significant differences were found between two groups in terms of time to first flatus, time to faeces, time to off bed activities, time to liquid diet, and time to soft diet (*P* > 0.05). Similarly, postoperative hospital stay did not differ between two groups (*P* = 0.900).

### 3.4. Postoperative Complications

Postoperative morbidities were similar in both groups (nonobese versus obese, 23.3% versus 23.8%, *P* = 0.931), as shown in Table 2. Obese patients seemed to experience more anastomotic leakage (6.3% versus 5.2%, *P* = 0.724), ileus (7.9% versus 6.0%, *P* = 0.580), and wound infection (7.9% versus 4.0%, *P* = 0.194), but this trend was not significant. Regarding the severity and degree of postoperative morbidity, major and minor complication rates were comparable between both groups (*P* = 0.133, *P* = 0.540).

Univariate analysis showed that age (*P* = 0.040), American Society of Anesthesiologists (ASA) score (*P* = 0.002), surgical type (*P* < 0.001), and diverting stoma (*P* = 0.002) were risk factors for postoperative complication (Table 3). Multivariate analysis demonstrated that higher ASA score (ASA 2: OR = 2.755, *P* = 0.012; ASA 3: OR = 6.274, *P* = 0.036) and abdominoperineal resection (OR = 4.972, *P* < 0.001) were independently associated with postoperative complications, and diverting stoma was a protective factor for postoperative complications (OR = 0.527, *P* = 0.037).

### 3.5. Oncological Safety

The proximal and distal lengths of the resection specimen (nonobese versus obese: proximal: 14.9 ± 3.5 cm versus 14.8 ± 1.9 cm, distal: 3.2 ± 1.2 cm versus 3.0 ± 1.1 cm) were not significantly different (*P* > 0.05), as shown in Table 4. Positive CRM rates for obese and nonobese patients were 1.2% and 3.2%, but the difference was not significant. The number of lymph node retrieved was comparable for both groups (*P* = 0.107). With respect to the tumor response to nCRT, no differences were found among the two groups regarding pathological TNM stage, pCR, and rectal cancer regression grade (*P* > 0.05).

**Figure 2:**
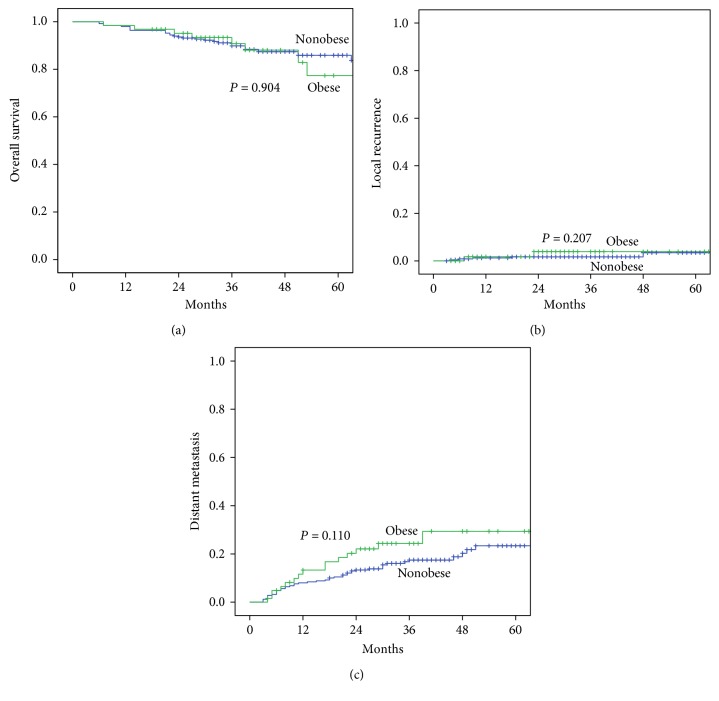
(a) Overall survival, (b) cumulative local recurrence, and (c) cumulative distant metastasis rate between nonobese and obese groups.

With a median follow-up time of 55 months (ranging 20–102 months), no significant difference was found in the 5-year overall survival rate between the two groups (nonobese versus obese: 85.9% versus 77.4%; *P* = 0.904), as presented in Figure 2(). The 5-year local recurrence rate (nonobese versus obese: 3.5% versus 3.9%; *P* = 0.207) was similar in the two groups. The 5-year distant metastasis rate was slightly higher in the obese group, but the difference was not significant (nonobese versus obese: 23.4% versus 29.4%; *P* = 0.110).

## 4. Discussion

The present study is the first to compare the surgical outcomes of laTME after nCRT in relation to obesity, using the Asian definition (BMI ≥ 25 kg/m^2^). The result demonstrates that obesity does not increase surgical morbidity or mortality during laTME and does not jeopardize short- and long-term oncological outcomes.

During rectal resection after CRT, the downsizing and downstaging of large tumor can improve exposure of the surgical field in the narrow pelvic cavity; however, tissue edema and vascularity associated with nCRT may hamper dissection of the tissue [10, 26]. The technical complexity of laTME is exacerbated in obese patients due to poor exposure of the operation field, difficulties in dissection, mobilization and ligation, more operative time required, and difficulties in maneuvering instruments in a restricted intraabdominal space [3, 5, 27]. It remains unclear whether these negative factors are capable of counteracting the advantages of laparoscopic surgery. In a meta-analysis of 8 studies including 2181 rectal cancer patients, laparoscopic procedure was more difficult in obese patients, together with an increase in the conversion rate and operative time [28]. When performing laTME after CRT, we often experienced difficulties in dissecting while recognizing a right plane of TME and were hampered by edema and exudates when utilizing ultrasonic dissectors, which contributed to a longer operative time. Obese patients did not suffer from increased estimated blood loss. The conversion rate in the current study (nonobese versus obese, 3.1% versus 6.3%) was comparable with 2.8% to 15.5% in the literature [29–31]. It was reported that tumor fixation, obesity, and intra-abdominal adhesions were the most common reasons for conversion during laparoscopic rectal resection [32]. In our series, procedures were performed by the same surgical team, which has experience with more than 3000 cases of laparoscopic colorectal surgeries since September, 2000 [19]. Our low conversion rate can be attributed to the high volume and extensive surgical experience of our specialized center. In our experience, laTME in obese patients with LARC after nCRT is feasible when performed by specialist surgeons.

In laparoscopic rectal surgery, there were some additional tricks in our center. The most important point was fully exposing the operation field. The patient was positioned at a 30-degree Trendelenburg and 15-degree right lateral tilt position. Gravity was utilized to handle the intestinal loops out of the operative field. Gauze was folded to protect the small bowel loops, especially when IMA high ligation was performed. We preferred to use fenestrated nontraumatic Babcock forceps to manipulate the bulky bowel mesentery, which was vulnerable to laceration and bleeding. In female patients, anterior exposure was achieved by hanging the uterus with a percutaneously inserted silk suture with a straight needle. Circumferential sharp dissection within the “holy plane” is fundamental to rectal cancer resection [33]. In our experience, maintaining good and constant tension contributes to reach the extremes of the pelvis. Furthermore, the pelvic autonomic nerve preservation is improved laparoscopically because of a magnified view of laparoscopic surgery [34]. Unfortunately, sexual and urinary functional data in our series were incomplete and thus could not be assessed in this study.

There is a concern regarding whether laparoscopic rectal resection can achieve adequate oncological clearance in obese patients. It was reported that higher BMI was an obstacle to perform proper lymph node dissection and the number of retrieved lymph nodes could be affected by obesity [35]. Additionally, the most significant reduction of mean retrieved lymph node numbers in rectal cancer was observed in obese patients with a short specimen length [35]. Nevertheless, considering the current recommendation of 12 lymph nodes or more for accurate staging [36], the average lymph nodes retrieved in the present study were acceptable, and no significant difference in specimen length was found between two groups. Additionally, significant prognostic factors in rectal cancer surgery, such as distal and circumferential resection margin involvement, were comparable, when compared with nonobese patients.

A phase 3 study (CAO/ARO/AIO-04), which integrated oxaliplatin into standard fluorouracil-based combined modality treatment, has reported that intensification of CRT was feasible and led to a higher pCR rate [14–16]. It has been noted that nCRT may result in significant morbidity, such as anastomotic leakage and wound infection [13, 37, 38]. Whether obesity will increase the risk of postoperative morbidity during laparoscopic resection after nCRT remains unanswered. In a recent meta-analysis, obese patients had a higher rate of minor complications after laparoscopic colorectal surgery, including ileus, wound infection, and respiratory events and comparable major complications, such as anastomotic leakage, which required surgical and endoscopic interventions [28]. Our study did not demonstrate an increase in overall morbidity in obese patients compared with nonobese patients. Also, the individual complication rate, including anastomotic leakage, ileus, and wound infection, did not differ between the two groups. Previous study found male sex, obesity, and preoperative radiation to be associated with postoperative complications after laparoscopic surgery for rectal cancer [31, 39]. In the current study, no association between BMI and postoperative morbidity was identified. Multivariate analysis showed that higher ASA score and abdominoperineal resection were independently associated with postoperative complications. These results suggested that obesity does not affect surgical safety in laparoscopic rectal resection for LARC patients following nCRT.

Transanal total mesorectal excision (taTME), an emerging surgical technique, has been described as a good solution for a male, with a narrow pelvis, obese patient with rectal cancer [40, 41]. However, whether taTME could achieve the standard of TME in the medium rectal cancer and part of low rectal cancer remains controversial, when compared with conventional laTME [42]. Additionally, comparable technical success of taTME might not be achieved in low-volume centers until this technique is fully accepted by surgeons [43].

There are several limitations in our study. First, because BMI is relatively lower in Asians than in non-Asians [44], obesity in this study was defined using IOTF classification for Asians (BMI ≥ 25 kg/m^2^). Second, Asian populations have greater visceral adiposity, and the impact of visceral fat in the field of colorectal surgery has been discussed mainly in Asian populations. BMI may be an imperfect marker for visceral obesity. Unfortunately, due to the retrospective nature of our study, the impact of visceral fat was not evaluated. We intended to evaluate the impact of visceral fat on surgical outcome of laparoscopic TME in LARC patients following nCRT in further studies. Third, this study is based on a single institutional retrospective analysis and subject to an inherent selection bias. Another limitation is that we did not conduct subgroup analysis of underweight patients owing to an insufficient number of samples in our series. We intend to explore these questions in the coming future studies.

## 5. Conclusion

Obesity does not affect the surgical and oncological outcomes of laparoscopic rectal resection after nCRT. LaTME may be feasible and safe to obese patients with LARC after nCRT in a high-volume center with sufficient experience. Further studies are needed to confirm the above findings.

## Figures and Tables

**Table 1:** Clinicopathological characteristics between nonobese and obese groups in locally advanced rectal cancer patients following nCRT.

Characteristics	BMI < 25 (*n* = 249)	BMI ≥ 25 (*n* = 63)	*P* value
Sex			0.085
Male	157 (63.1)	47 (74.6)	
Female	92 (36.9)	16 (25.4)	
Age (years)	54.9 ± 12.0	54.7 ± 9.1	0.861
BMI (kg/m^2^)	21.7 ± 2.2	26.9 ± 2.8	<0.001
ASA score			0.658
1	164 (65.9)	45 (71.4)	
2	78 (31.3)	17 (27.0)	
3	7 (2.8)	1 (1.6)	
Distance from the anal verge (cm)	5.9 ± 2.1	5.9 ± 1.8	0.948
Gross type			0.843
Expanding	56 (22.5)	16 (25.4)	
Ulcering	178 (71.5)	44 (69.8)	
Infiltrating	15 (6.0)	3 (4.8)	
Histopathology			0.100
Adenocarcinoma	227 (91.2)	53 (84.1)	
Mucinous or signet ring adenocarcinoma	22 (8.8)	10 (15.9)	
Tumor differentiation			0.196
Well or moderately differentiated	214 (85.9)	50 (79.4)	
Poorly differentiated and others^a^	35 (14.1)	13 (20.6)	
Clinical T stage			0.230
T3	57 (22.9)	19 (30.2)	
T4	192 (77.1)	44 (69.8)	
Clinical N stage			0.580
N0	15 (6.0)	5 (7.9)	
N+	234 (94.0)	58 (92.1)	
Preoperative CEA level (ng/ml)			0.650
<5	216 (86.7)	56 (88.9)	
≥5	33 (13.3)	7 (11.1)	

Data are expressed as number (%) or as median ± standard deviation, where appropriate. ^a^Including mucinous and signet cell carcinoma. nCRT: neoadjuvant chemoradiotherapy; BMI: body mass index; ASA: American Society of Anesthesiology; CEA: carcinoembryonic antigen.

**Table 2:** Perioperative outcomes between nonobese and obese groups in locally advanced rectal cancer patients following nCRT.

Variables	BMI < 25 (*n* = 249)	BMI ≥ 25 (*n* = 63)	*P* value
Operative time (min)	207.9 ± 43.9	224.3 ± 38.8	0.004
Estimated blood loss (ml)	63.2 ± 87.6	73.6 ± 59.1	0.265
Conversion	9 (3.6)	4 (6.3)	0.332
Surgical procedure			0.497
Sphincter-preserving surgery	222 (89.2)	58 (92.1)	
Abdominoperineal resection	27 (10.8)	5 (7.9)	
Diverting stoma	127 (51.0)	34 (54.0)	0.674
Postoperative hospital stay	9.0 ± 6.1	8.9 ± 6.7	0.900
Time to flatus (days)	1.9 ± 0.9	1.9 ± 0.7	0.751
Time to faeces (days)	3.1 ± 1.7	2.9 ± 1.5	0.412
Time to off bed activities (days)	1.9 ± 0.8	1.9 ± 0.9	0.829
Time to liquid diet (days)	1.9 ± 1.0	1.8 ± 0.9	0.297
Time to soft diet (days)	4.5 ± 2.2	4.3 ± 1.7	0.463
Overall morbidity	58 (23.3)	15 (23.8)	0.931
Postoperative complications^a^			
Anastomotic leakage	13 (5.2)	4 (6.3)	0.724
Ileus	15 (6.0)	5 (7.9)	0.580
Wound infection	10 (4.0)	5 (7.9)	0.194
Sepsis	3 (1.2)	1 (1.6)	0.809
Acute urinary retention	5 (1.6)	2 (3.2)	0.576
Respiratory complications	10 (4.0)	4 (6.3)	0.424
Others^b^	9 (3.6)	4 (6.3)	0.332
Reoperation	2 (0.8)	1 (1.6)	0.569
Grade of morbidity			
Minor	46 (18.5)	17 (27.0)	0.133
Major	25 (10.0)	8 (12.5)	0.540

^a^Some patients experienced more than one complication. ^b^Including cardiovascular events, cerebrovascular events, deep vein thrombosis, and chyle leakage. Data are expressed as number (%) or as median ± standard deviation, where appropriate. nCRT: neoadjuvant chemoradiotherapy.

**Table 3:** Univariate and multivariate analysis of perioperative complications for patients with locally advanced rectal cancer after nCRT.

Variables	Complication (−)	Complication (+)	Univariate	Multivariate		
	*n* = 239	*n* = 73	*P* value	OR	95% CI	*P* value
Age (year)	54. 1 ± 11.0	57.5 ± 12.5	0.040	0.268	0.963–1.030	0.136
Gender			0.529			
Male	156 (65.3)	48 (65.8)				
Female	83 (34.7)	25 (34.2)				
BMI (kg/m^2^)			0.525			
<25	191 (79.9)	58 (79.5)				
≥25	48 (20.1)	15 (20.5)				
ASA score			0.002			
1	172 (72.0)	37 (50.7)		1		
2	63 (26.4)	32 (43.8)		2.755	1.253–6.060	0.012
3	4 (1.7)	4 (5.5)		6.274	1.123–35.039	0.036
Previous laparotomy history	25 (10.5)	7 (9.6)	0.829			
Tumor distance from the anal verge (cm)	6.0 ± 1.9	5.6 ± 2.2	0.129			
Tumor diameter (cm)	2.9 ± 1.0	2.9 ± 1.1	0.491			
Radiation dose (cGy)	4869.8 ± 443.2	4908.5 ± 336.5	0.426			
Interval to surgery (weeks)	8.0 ± 2.0	8.4 ± 2.4	0.229			
Surgical type			<0.001			
Sphincter-preserving surgery	226 (94.6)	54 (74.0)		1		
Abdominoperineal resection	13 (5.4)	19 (26.0)		4.972	2.178–11.349	<0.001
Diverting stoma	135 (56.4)	26 (35.6)	0.002	0.527	0.289–0.963	0.037
Conversion	10 (4.2)	3 (4.1)	0.639			
Operative time (min)	209.0 ± 41.1	218.5 ± 49.6	0.143			
Estimated blood loss (ml)	64.0 ± 88.3	69.7 ± 60.1	0.606			
Pathological TNM stage			0.299			
0	40 (16.7)	18 (24.7)				
I	61 (25.5)	15 (20.5)				
II	72 (30.1)	17 (23.3)				
III	66 (27.6)	23 (31.5)				
Rectal cancer regression grade			0.967			
1	126 (52.7)	39 (53.4)				
2	91 (38.1)	28 (38.4)				
3	22 (9.2)	6 (8.2)				

Data are expressed as number (%) or as median ± standard deviation, where appropriate. OR: odds ratio; CI: confidential interval; BMI: body mass index; ASA: American Society of Anesthesiologists. ^a^Including mucinous and signet cell carcinoma.

**Table 4:** Oncological clearance between nonobese and obese groups in locally advanced rectal cancer patients after nCRT.

Characteristics	BMI < 25 (*n* = 249)	BMI ≥ 25 (*n* = 63)	*P* value
Length of resection margin (cm)			
Proximal	14.9 ± 3.5	14.8 ± 1.9	0.864
Distal	3.2 ± 1.2	3.0 ± 1.1	0.172
CRM involvement	3 (1.2)	2 (3.2)	0.266
Tumor clearance			0.382
R0	242 (97.2)	59 (93.7)	
R1	4 (1.6)	2 (3.2)	
R2	3 (1.2)	2 (3.2)	
No. of lymph node retrieved	12.7 ± 8.2	11.4 ± 4.8	0.107
Lymph node ratio^∗^	0.08 (0–0.86)	0.04 (0–0.47)	0.278
ypUICC stages			0.617
0	48 (18.3)	10 (15.9)	
I	59 (23.7)	17 (27.0)	
II	68 (27.3)	21 (33.3)	
III	74 (29.7)	15 (23.8)	
Tumor grade			0.196
G1 + G2	214 (85.9)	50 (79.4)	
G3 + GX	35 (14.1)	13 (20.6)	
pCR	48 (19.3)	10 (15.9)	0.535
RCRG			0.221
I	137 (55.0)	28 (44.4)	
II	89 (35.7)	30 (47.6)	
III	23 (9.2)	5 (7.9)	

Data are expressed as number (%) or as median ± standard deviation, where appropriate.^∗^Data are expressed as median (range) and analyzed with the Mann–Whitney *U* test. nCRT: neoadjuvant chemoradiotherapy; CRM: circumferential resection margin; pCR: pathological complete response; RCRG: rectal cancer regression grade: RCRG 1, sterilization or only microscopic foci of adenocarcinoma remaining, with marked fibrosis; RCRG 2, marked fibrosis but macroscopic disease present; RCRG 3, little or no fibrosis, with abundant macroscopic disease.
